# Patient-related risk factors for outlet obstruction in diverting loop ileostomy following minimally invasive rectal cancer surgery

**DOI:** 10.1007/s10151-025-03258-5

**Published:** 2025-12-24

**Authors:** M. Ishii, A. Hamabe, K. Okita, T. Nishidate, K. Okuya, E. Akizuki, A. Noda, M. Miyo, R. Miura, M. Toyota, K. Okamoto, I. Takemasa

**Affiliations:** 1 Department of Surgery, Division of Gastroenterological Surgery, Sapporo Medical University, S1 W16, Chuo-ku, Sapporo, 060-8543 Japan; 2 Department of Gastroenterological Surgery, Higashi Sapporo Hospital, Hokkaido, Japan; 3 Department of Gastroenterological Surgery, Graduate School of Medicine, Osaka University, Osaka, Japan; 4 Department of Gastroenterological Surgery, Otaru Ekisaikai Hospital, Hokkaido, Japan; 5 Department of Gastroenterological Surgery, JR Sapporo Hospital, Hokkaido, Japan; 6 Department of Anorectal Surgery, Sapporo Ishiyama Hospital, Hokkaido, Japan; 7 Department of Gastroenterological Surgery, Osaka International Cancer Institute, Osaka, Japan; 8 Department of Gastroenterological Surgery, Osaka International Medical and Science Center, Osaka Keisatsu Hospital, Osaka, Japan

**Keywords:** Outlet obstruction, Ileostomy, Rectal cancer, Risk factors, Visceral fat, Abdominal muscles

## Abstract

**Introduction:**

Anastomotic leakage (AL) is a serious complication after rectal cancer resection, often mitigated by diverting loop ileostomy. However, outlet obstruction remains a significant concern, potentially prolonging hospitalization and requiring reintervention. While surgical risk factors have been explored, patient-specific anatomical factors are less well understood. This study aimed to identify patient-related risk factors for outlet obstruction and evaluate a preventive surgical modification in high-risk patients undergoing laparoscopic and robotic rectal cancer surgeries.

**Methods:**

This retrospective study included 318 patients who underwent laparoscopic or robotic rectal resection with a diverting loop ileostomy. Risk factors were assessed in a control cohort (April 2015–February 2020), followed by a modified ileostomy technique in a validation cohort (March 2020–December 2024).

**Results:**

Increased rectus abdominis muscle thickness (TAM) and larger visceral fat area (AVF) were independent risk factors for outlet obstruction (*p* = 0.037 and *p* = 0.041, respectively). Patients with both factors had the highest incidence (52.6%). The modified technique significantly reduced obstruction among high-risk patients (*p* = 0.003) without increasing parastomal hernia rates.

**Conclusions:**

TAM and AVF are independent predictors of outlet obstruction. A tailored fascial modification reduced obstruction in high-risk patients, supporting the value of preoperative anatomical assessment in surgical planning.

## Introduction

Anastomotic leakage (AL) is a serious and potentially life-threatening complication following rectal cancer resection. AL increases postoperative morbidity and mortality and is associated with a higher risk of local recurrence [1, 2]. To reduce the risk of severe AL, diverting stomas are frequently constructed [3]. Among the two primary types of stoma construction methods (ileostomy and colostomy), ileostomy is generally preferred due to its relative ease of creation and reversal compared with colostomy [4–6]. However, ileostomy carries its own risks, including skin irritation, parastomal hernia, stenosis, prolapse, and notably, outlet obstruction [7–9]. Outlet obstruction is a clinically significant complication that can result in vomiting, aspiration, prolonged hospitalization, and even reoperation [10–12].

Although prior studies have implicated factors such as postoperative adhesion, stomal site stenosis, and ileal twisting as causes of outlet obstruction [7, 13, 14], the widespread adoption of minimally invasive surgery (MIS), including laparoscopic and robotic approaches, has changed the surgical landscape. MIS is associated with fewer adhesions compared with open surgery [15–19], yet outlet obstruction still occurs in 5.4–27.3% of MIS cases [20–23]. These findings underscore the need to reevaluate risk factors for outlet obstruction in the context of contemporary surgical techniques. MIS has become the standard approach for rectal cancer surgery and is routinely performed in our department. Compared with open surgery, MIS is associated with fewer postoperative adhesions, which may influence the mechanisms underlying outlet obstruction. As MIS continues to expand in clinical practice, identifying patient-related risk factors and optimizing stoma construction techniques in this context is of increasing clinical importance.

Importantly, while several risk factors have been identified (e.g., tight fascial closure, intestinal torsion), patient-related anatomical features such as rectus abdominis thickness and visceral fat area have not been thoroughly investigated. Given the potential clinical implications, this study aimed to identify patient-specific risk factors associated with outlet obstruction and to assess the utility of a modified ileostomy technique designed to reduce its incidence.

## Methods

### Patients

**Fig. 1:**
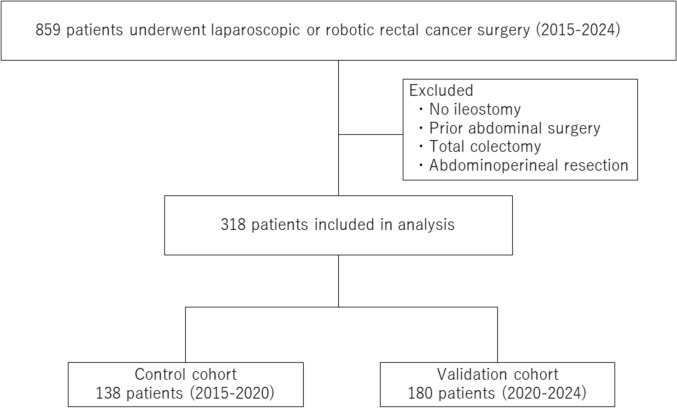
Flowchart illustrating patient selection. Among 859 patients who underwent laparoscopic or robotic rectal cancer surgery between 2015 and 2024, 318 were included in the final analysis after excluding those without an ileostomy, those with prior abdominal surgery, total colectomy, or abdominoperineal resection. Patients were divided into a control cohort (*n* = 138, 2015–2020) and a validation cohort (*n* = 180, 2020–2024)

Between April 2015 and December 2024, 859 patients underwent laparoscopic or robotic rectal resection for rectal cancer at our institution. A total of 859 patients underwent laparoscopic or robotic rectal cancer surgery during the study period. Patients were excluded if they did not receive a diverting loop ileostomy, had a history of abdominal surgery, inflammatory bowel disease (including those who had undergone total colectomy), or received abdominoperineal resection (APR). Ultimately, 318 patients were included in the analysis. A flowchart summarizing the inclusion and exclusion criteria is presented in Fig. 1. Among these, 318 consecutive patients who underwent diverting loop ileostomy were retrospectively analyzed. Risk factors for outlet obstruction were first assessed in a control cohort treated between April 2015 and February 2020. On the basis of these findings, a modified ileostomy construction technique was implemented, and its impact on the incidence of outlet obstruction was evaluated in a validation cohort from March 2020 to December 2024.

### Analysis

**Fig. 2:**
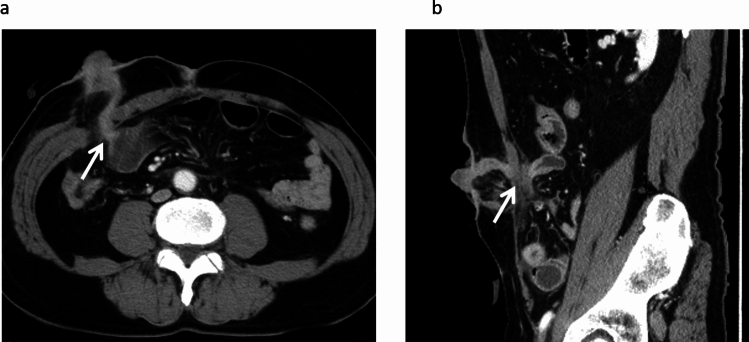
Representative computed tomographic image of stoma outlet obstruction. **a** Axial view showing outlet obstruction at the site where the stoma penetrates the abdominal wall. **b** Sagittal views showing outlet obstruction at the same site

Outlet obstruction was defined as intestinal dilatation with impaired contrast passage on computed tomography (CT) and resolution following decompression via tubing of the proximal ileostomy limb, in line with criteria used in previous studies (Fig. 2) [22, 24]. Parastomal hernia was defined as a protrusion of intraabdominal contents through the abdominal wall defect created during stoma formation, in accordance with the European Hernia Society classification [25]. High-output stoma was defined as a daily output of ≥ 2000 mL for three consecutive days on the basis of criteria indicating a risk of dehydration and renal dysfunction [10]. Patients were categorized into two groups: those who developed outlet obstruction and those who did not. The following patient- and tumor-related characteristics were assessed: age, sex, body mass index (BMI), American Society of Anesthesiologists (ASA) score, thickness of the rectus abdominis muscle (TAM), thickness of subcutaneous fat (TSF), area of subcutaneous fat (ASF), area of visceral fat (AVF), stoma-related complications (high-output stoma, parastomal hernia, stoma prolapse, or stoma retraction), and surgical parameters. The primary aim of this study was to identify anatomical risk factors associated with outlet obstruction. On the basis of these findings, a modified ileostomy technique was introduced as a targeted intervention rather than a variable for comparative analysis. Therefore, multivariate analysis was limited to the control cohort to avoid confounding by surgical technique. To assess the reproducibility of the modified ileostomy technique, we retrospectively measured the length of the fascial opening at the stoma site using sagittal CT images obtained prior to stoma closure. Measurements were performed in both the control and validation cohorts, including a subgroup analysis of high-risk patients.

**Fig. 3:**
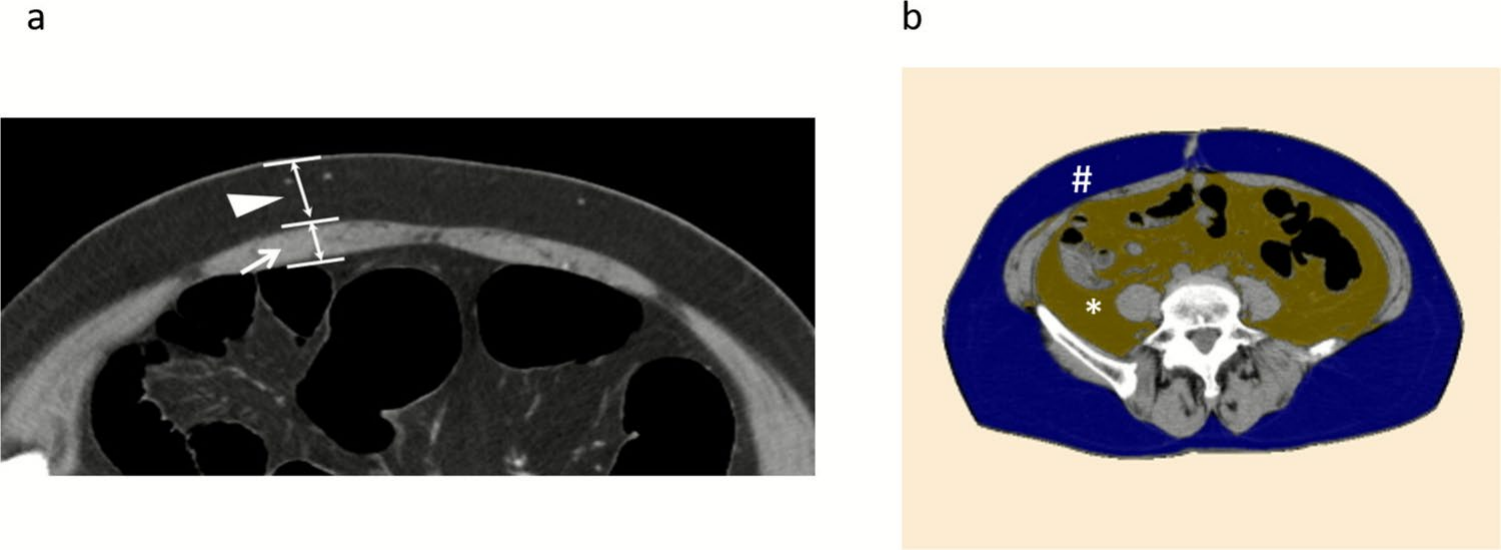
Measurement of TAM, TSF, AVF, and ASF. **a** TAM and TSF were measured as vertical distances at the planned ileostomy site on preoperative CT. **b** ASF and AVF were calculated at the level of the umbilicus using the SYNAPSE VINCENT 3D image analysis system. Arrow: TAM distance; arrowhead: TSF distance; #, blue area: ASF; *, yellow area: AVF. *TAM* thickness of the rectus abdominis muscle, *TSF* thickness of the subcutaneous fat, *AVF* area of the visceral fat, *ASF* area of the subcutaneous fat

TAM and TSF were measured preoperatively on CT as vertical distances to the abdominal wall at the planned ileostomy site (Fig. 3A). ASF and AVF were calculated at the level of the umbilicus using the SYNAPSE VINCENT (Fujifilm Medical, Tokyo, Japan), version 6.4 three-dimensional (3D) image analysis system (Fig. 3B). The segmentation was performed using the software’s standard threshold settings for adipose tissue (−200 to −50 Hounsfield units for subcutaneous and visceral fat). The software automatically calculated the area (cm^2^) by summing the pixels within this range. CT images were acquired with a slice thickness of 5 mm, and measurements were taken from axial slices. All measurements were conducted retrospectively using a standardized protocol and were reviewed by two independent observers to ensure consistency.

### Surgical procedure

All patients underwent standard laparoscopic or robotic rectal cancer resection at our institution. Following the placement of an umbilical port, pneumoperitoneum was established with CO_2_ insufflation at 10 mmHg, and four to five additional ports were placed. The procedure was performed using a multiport technique. Mobilization was achieved using a medial-to-lateral approach. The distal rectum was transected intracorporeally using a laparoscopic or robotic linear stapler, or via transanal full-thickness tractotomy. Following tumor resection, the anastomosis was completed using the end-to-end double-stapling technique. In cases requiring intersphincteric resection (ISR), hand-sewn anastomosis was performed from the anal side. A total of 15 surgeons participated in the stoma creation procedures during the study period: 7 in the control cohort and 8 in the validation cohort. All surgeons employed the same ileostomy construction method, with the only variation being the length of the fascial opening, which was adjusted in the validation cohort on the basis of anatomical risk factors.

To minimize interoperator variability, the surgical protocol for stoma creation was standardized across all participating surgeons. This included consistent procedures for fascial incision, stoma elevation without rotation, and fixation to the anterior rectus sheath. Although minor individual differences may exist, the core technique remained uniform throughout the study period, thereby reducing the likelihood of operator-related bias in the outcomes. Before implementing the modified technique in the validation cohort, all participating surgeons underwent structured training sessions, including intraoperative demonstrations and protocol briefings. This ensured uniform application of the modified approach and minimized interoperator variability.

### Creation of diverting loop ileostomy

**Fig. 4:**
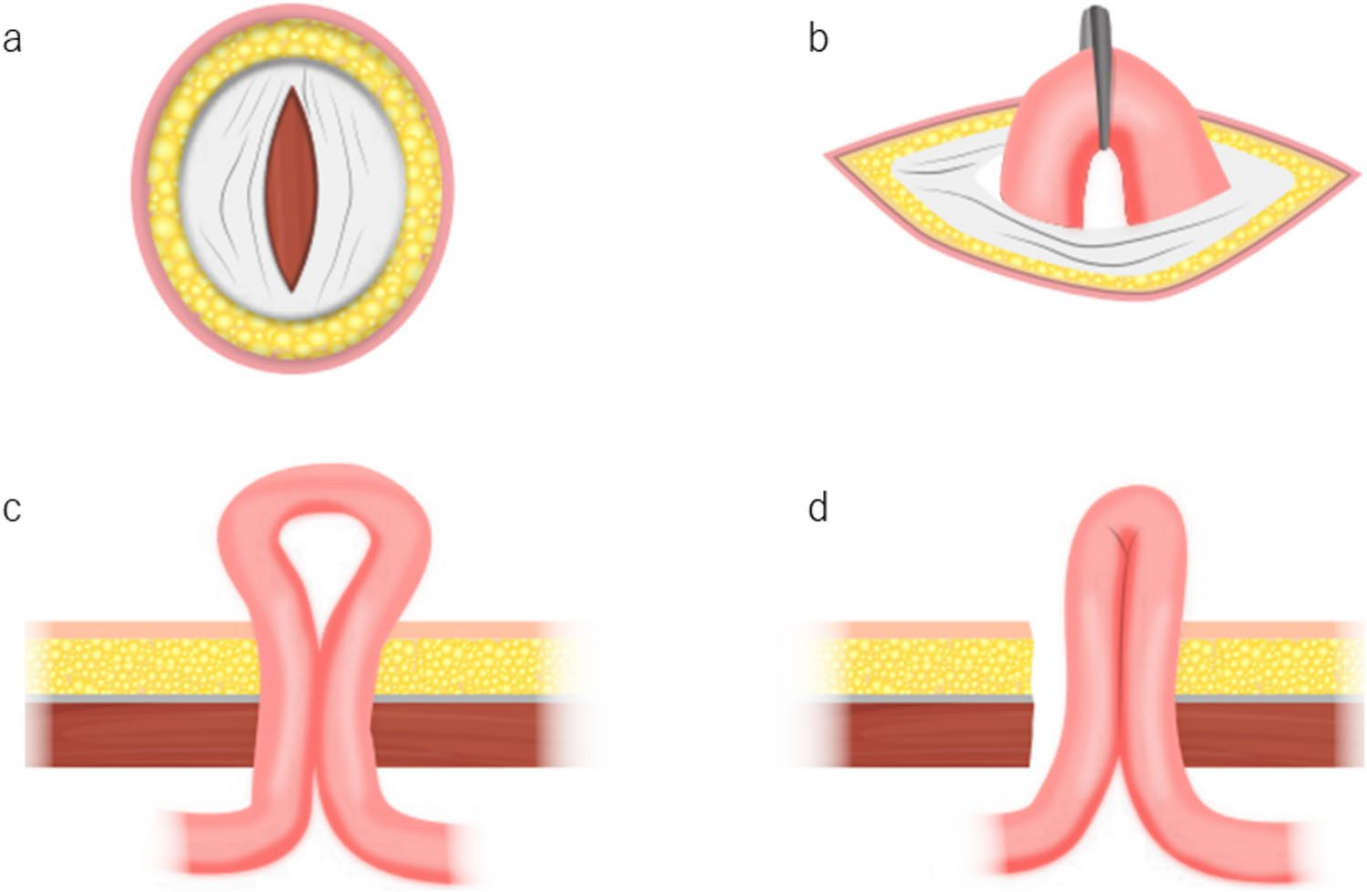
Comparison of ileostomy construction techniques

The stoma site was marked preoperatively according to the Cleveland Clinic guidelines [26]. A straight skin incision was made at the selected site. The stoma was created at the designated location rather than through a previously used port. Although patient body habitus and intraoperative considerations occasionally required adjustments, the standardized ileostomy construction technique was consistently applied regardless of the final stoma position. In the control cohort, the rectus abdominis muscle was split, and the peritoneum was opened to approximately two finger widths. In the validation cohort, following ileostomy elevation, the abdominal wall was dissected as widely as possible by inserting a finger between the intestinal loop and fascia to prevent torsion. The ileostomy was created 30–35 cm proximal to the ileocecal valve, ensuring natural elevation without rotation. The proximal and distal ileostomy limbs were approximately 2.5 cm and 1.0 cm in height, respectively. The seromuscular layer of the intestine was fixed to the anterior sheath of the rectus abdominis using two absorbable sutures. A schematic comparison of the conventional and modified ileostomy techniques is shown in Fig. 4. The modified approach adjusted the fascial opening to the width of one finger after extracorporeal elevation of the bowel loop, aiming to minimize outlet obstruction in anatomically high-risk patients.

In the conventional technique, the rectus abdominis muscle and fascia are incised to a width of approximately two fingers regardless of patient body habitus, and the bowel loop is elevated. In the modified technique, the bowel loop is first elevated extracorporeally, and the fascial incision is then adjusted to allow passage of only one finger between the bowel and the fascia.

(a, c) Conventional technique:

(a) Fascial incision with two-finger width.

(c) Cross-sectional view of the abdominal wall showing the conventional fascial opening.

(b, d) Modified technique:

(b) Fascial adjustment to one-finger width following extracorporeal elevation.

(d) Cross-sectional view of the abdominal wall showing the modified fascial opening.

### Indications for diverting stoma construction

A diverting ileostomy was performed in all patients with anastomoses located within 5 cm of the anal verge. Additional indications included the presence of at least two of the following factors: (i) male sex, (ii) tumor size ≥ 4 cm, (iii) three or more staple firings, (iv) BMI ≥ 25, (v) preoperative bowel obstruction, and (vi) preoperative chemoradiotherapy.

### Perioperative management

Preoperative bowel preparation consisted of mechanical and oral antibiotic cleansing. Patients received 100 g of magnesium citrate in 400 mL of water 2 days before surgery, followed by 3 g of kanamycin and 750 mg of metronidazole on the day before surgery. Postoperatively, patients resumed drinking water on postoperative day 1, received nutritional support on day 2, and initiated oral intake thereafter.

### Statistical analysis

Patient demographics and surgical variables were summarized as frequencies (%) or medians (ranges). Between-group comparisons were performed using Fisher’s exact test, the Mann–Whitney *U* test, or the Student’s *t* test, as appropriate. Variables with *p* < 0.1 in univariate analysis were entered into multivariate logistic regression to calculate adjusted odds ratios. Cutoff values were determined using receiver operating characteristic (ROC) curve analysis. Statistical analyses were performed using EZR (version 2.13.0; Jichi Medical University, Saitama, Japan), a statistical software package based on R [27]. Statistical significance was set at *p* < 0.05.

## Results

Patient characteristics and surgical variables, including percentage distributions, are summarized in Table 1. Among the 318 patients, 198 (62.2%) were male, with a median age of 62 years (range 27–89 years) and a median BMI of 23.5 kg/m^2^ (range 13.3–37.6 kg/m^2^). Neoadjuvant therapy was administered to 175 patients (55.0%). Surgical procedures included high anterior resection (3 patients, 1.0%), low anterior resection (192 patients, 60.4%), and ISR (123 patients, 38.6%). Laparoscopic surgery was performed on 75 patients (23.6%), and robotic surgery on 241 (76.4%).

**Table 1:** Patient, tumor, and surgical characteristics

Characteristics	*n* = 318
Patient characteristics	
Sex (male/female)	198 (62.2%)/120 (37.8%)
Age (years)	62 (27–89)
BMI (kg/m^2^)	23.5 (13.3–37.6)
TAM (mm)	9.7 (3.8–20.4)
TSF (mm)	16.4 (1.5–38.8)
AVF (cm^2^)	101.1 (2.1–285.5)
ASF (cm^2^)	121.9 (2.4–444.6)
ASA (1/2/3/4/)	91 (28.6%)/209 (65.7%)/18 (5.7%)
Tumor characteristics	
cT (1/2/3/4)	34 (10.7%)/77 (24.2%)/186 (58.5%)/21 (6.6%)
cN (0/1/2/3)	221 (69.8%)/57 (17.9%)/18 (5.7%)
cM (0/1)	291 (91.5%)/27 (8.5%)
cStage (1/2/3/4)	99 (31.1%)/76 (23.9%)/27 (8.5%)
Preoperative therapy	
Neoadjuvant treatment (yes/no)	175 (55.0%)/153 (45.0%)
Surgical procedure	
LAR/ISR	195 (60.4%)/123 (38.6%)
Surgical approach	
Laparoscopic/robotic	75 (23.6%)/241 (76.4%)
LLND (yes/no)	111 (34.9%)/207 (65.1%)
Operation time (min)	481 (175–1054)
Bleeding (mL)	10 (1–1245)
Outlet obstruction (yes/no)	23 (7.2%)/295 (92.8%)
High-output stoma (yes/no)	56 (17.6%)/262 (82.4%)
Parastomal hernia (yes/no)	27 (8.5%)/291 (91.5%)
Stoma prolapse (yes/no)	0 (0%)/318 (100%)
Stoma retraction (yes/no)	0 (0%)/318 (100%)

*BMI* body mass index, *TAM* thickness of the rectus abdominis muscle, *TSF* thickness of the subcutaneous fat, *AVF* area of the visceral fat, *ASF* area of the subcutaneous fat, *ASA* American Society of Anesthesiologists, *LAR* low anterior resection, *ISR* intersphincteric resection, *LLND* lateral lymphadenectomy

The median follow-up period was 186 days (range 27–1090 days), corresponding to the typical interval before stoma closure. Outlet obstruction occurred in 23 patients (7.2%). No patient required surgical intervention for outlet obstruction, and all cases resolved with tubing of the proximal ileostomy limb. High-output stoma was observed in 56 patients (17.6%) and parastomal hernia occurred in 27 patients (8.5%).

Patient characteristics by group are presented in Table 2. The control group had significantly longer operative times and more frequent lateral lymph node dissections (LLND). Robotic surgery was more commonly performed in the validation cohort. No significant differences were observed in other parameters between groups.

**Table 2:** Patient, tumor, and surgical characteristics in each group

	Control (*n* = 138)	Validation (*n* = 180)	*p*-Value
Characteristics			
Patient characteristics			
Sex (male/female)	89 (64.4%)/49 (35.6%)	108 (60.0%)/72 (40.0%)	0.353
Age (years)	62 (27–85)	64 (31–89)	0.841
BMI (kg/m^2^)	23.7 (15.5–36.4)	23.3 (13.3–37.6)	0.689
TAM (mm)	9.65 (5.5–16.1)	9.70 (3.8–20.4)	0.629
TSF (mm)	15.6 (4.9–36.4)	14.5 (1.5–38.8)	0.617
AVF (cm^2^)	98.2 (2.1–285.5)	101.1 (16.0–250.1)	0.657
ASF (cm^2^)	130.9 (4.9–367.7)	113.3 (2.4–444.6)	0.244
ASA (1/2/3/4)	34 (24.6%))/93 (67.4%)/11 (8.0%)	57 (31.6%)/116 (64.4%)/7 (3.9%)	0.245
Tumor characteristics			
cT (1/2/3/4)	15 (10.9%)/32 (23.2%)/87 (63.0%)/4 (2.9%)	19 (10.6%)/52 (28.9%)/92 (51.1%)/17 (9.4%)	0.181
cN (0/1/2/3)	101(73.2%)/19 (13.8%)/10 (7.2%)/8 (5.8%)	120 (66.7%)/36 (20.0%)/8 (4.4%)/11 (6.1%)	0.523
cM (0/1)	126 (91.3%)/12 (8.7%)	165 (91.7%)/15 (8.3%)	0.901
cStage (0/1/2/3/4)	34 (35.8%)/61 (64.2%)/31 (72.1%)/12 (27.9%)	65 (54.2%)/55 (45.8%)/45 (75.0%)/15 (25.0%)	0.103
Preoperative therapy			
Neoadjuvant treatment (yes/no)	77 (55.8%)/61 (44.2%)	98 (51.6%)/92 (48.4%)	0.519
Surgical procedure			
LAR/ISR	76 (55.1%)/62 (44.9%)	119 (66.1%)/61 (33.9%)	0.049
Surgical approach			
Laparoscopic/robotic	67 (48.6%)/71 (51.4%)	8 (4.4%)/172 (95.6%)	< 0.001
Operation time (min)	425 (231–1088)	397 (175–1012)	< 0.001
Bleeding (mL)	10 (5–600)	10 (1–1245)	0.568
LLND (yes/no)	68 (49.3%)/70 (50.7%)	43 (23.9%)/137 (76.1%)	< 0.001
Outlet obstruction	15 (10.9%)/123 (89.1%)	8 (4.4%)/172 (95.6%)	0.047
High-output stoma (yes/no)	26 (18.9%)/112 (81.1%)	30 (16.7%)/150 (83.3%)	0.614
Parastomal hernia (yes/no)	11 (8.0%)/126 (92.0%)	16 (8.9%)/162 (91.1%)	0.763
Stoma prolapse (yes/no)	0 (0%)/138 (100%)	0 (0%)/180 (100%)	1.000
Stoma retraction (yes/no)	0 (0%)/138 (100%)	0 (0%)/180 (100%)	1.000

*BMI* body mass index, *TAM* thickness of the rectus abdominis muscle, *TSF* thickness of the subcutaneous fat, *AVF* area of the visceral fat, *ASF* area of the subcutaneous fat, *ASA* American Society of Anesthesiologists, *LAR* low anterior resection, *ISR* intersphincteric resection, *LLND* lateral lymphadenectomy

Table 3 presents the risk factors for outlet obstruction in the control cohort. In this cohort, outlet obstruction occurred in 15 patients (10.9%). BMI was significantly higher in the outlet obstruction group (*p* = 0.042). Both TAM and AVF were significantly greater in patients with outlet obstruction (*p* = 0.005 and *p* = 0.006, respectively). No significant differences were found in sex, age, ASA score, histopathology, preoperative therapy, surgical procedure, approach, TSF, ASF, operative time, estimated blood loss, LLND, parastomal hernia, high-output stoma, stoma prolapse, or stoma retraction.

**Table 3:** Univariate and multivariate analyses of risk factors for outlet obstruction

	*n* = 138		Univariate	Multivariate		
	Outlet obstruction (+) (*n* = 15)	Outlet obstruction (−) (*n* = 123)	*p*	Odds ratio	95% CI	*p*-Value
Characteristics						
Sex (male/female)	11 (73.3%)/4 (26.7%)	79 (64.2%)/44 (35.8%)	0.576			
Age (years)	69 (31–72)	65 (22–85)	0.597			
BMI (kg/m^2^)	25.1 (19.3–29.4)	22.6 (15.6–36.4)	0.042	0.917	0.854–1.390	0.493
TAM (mm)	11.5 (8.3–14.2)	9.5 (5.5–16.1)	0.005	1.355	1.016–1.805	0.037
TSF (mm)	28.9 (10.1–34.2)	28.3 (5.8–43.2)	0.536			
AVF (cm^2^)	142.3 (16.3–206.1)	95.7 (2.1–285.5)	0.006	1.019	1.000–1.040	0.041
ASF (cm^2^)	178.3 (35.1–291.7)	127.9 (8.5–359.2)	0.119			
ASA (1/2/3/4)	4 (28.6%)/10 (71.4%)/1	29 (25.7%)/84 (74.3%)/10	0.818			
Tumor characteristics						
cT (1/2/3/4)	4 (66.7%)/2 (33.3%)/8 (88.9%)/1 (11.1%)	11 (34.4%)/21 (65.6%)/87 (95.6%)/4 (4.4%)	0.297			
cN (0/1/2/3)	10 (83.3%)/2 (16.7%)/1 (33.3%)/2 (66.7%)	91 (84.3%)/17 (15.7%)/9 (60.0%)/6 (40.0%)	0.414			
cM (0/1)	14 (93.3%)/1 (6.7%)	112 (91.1%)/11 (8.9%)	0.827			
cStage (0/1/2/3/4)	6 (54.5%)/5 (45.5%)/3 (75.0%)/1 (25.0%)	29 (33.7%)/57 (66.3%)/26 (70.3%)/11 (29.7%)	0.390			
Preoperative therapy						
Neoadjuvant treatment (yes/no)	6 (40.0%)/9 (60.0%)	72 (58.5%)/51 (41.5%)	0.288			
Surgical procedure						
LAR/ISR	9 (60.0%)/6 (40.0%)	66 (53.7%)/57 (46.3%)	0.556			
Surgical approach						
Laparoscopic/robotic	9 (60.0%)/6 (40.0%)	58 (47.2%)/65 (52.8%)	0.248			
Operation time (min)	541 (294–454)	475 (231–1088)	0.975			
Bleeding (mL)	10 (5–515)	10 (5–600)	0.827			
LLND (yes/no)	7 (46.7%)/8 (53.3%)	62 (50.4%)/61 (49.6%)	0.785			
High-output stoma (yes/no)	26 (18.9%)/112 (81.1%)	30 (16.7%)/150 (83.3%)	0.614			
Parastomal hernia (yes/no)	11 (8.0%)/126 (92.0%)	16 (8.9%)/162 (91.1%)	0.763			

*TAM* thickness of the rectus abdominis muscle, *TSF* thickness of the subcutaneous fat, *AVF* area of the visceral fat, *ASF* area of the subcutaneous fat, *ASA* American Society of Anesthesiologists, *LAR* low anterior resection, *ISR* intersphincteric resection, *LLND* lateral lymphadenectomy, *CI* confidence interval

**Fig. 5:**
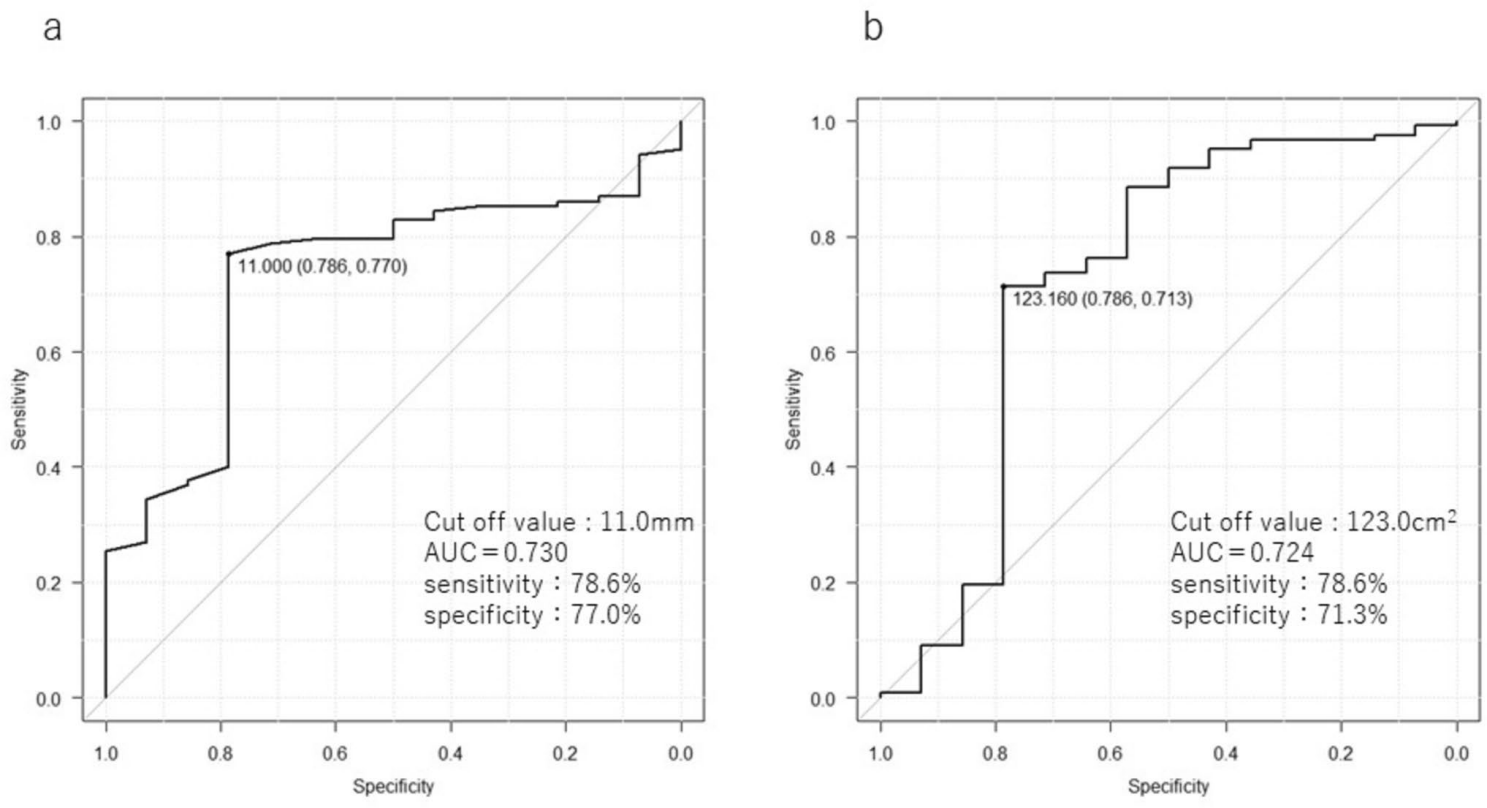
Cutoff values for TAM and AVF. **a** Cutoff value for TAM. **b** Cutoff value for AVF. *TAM* thickness of the rectus abdominis muscle, *AVF* area of the visceral fat, *AUC* area under the curve

Multivariate analysis identified TAM and AVF as independent risk factors for outlet obstruction: TAM (odds ratio 1.355; 95% confidence interval 1.016–1.805, *p* = 0.037) and AVF (odds ratio 1.019; 95% confidence interval 1.000–1.040, *p* = 0.041) (Table 3). Receiver-operating characteristic (ROC) analysis for TAM showed an area under the curve (AUC) of 0.73, with an optimal cutoff value of 11.0 mm (sensitivity: 78.6%, specificity: 77.0%). For AVF, the AUC was 0.724, with an optimal cutoff of 123.0 cm^2^ (sensitivity: 78.6%, specificity: 71.3%) (Fig. 5).

**Fig. 6:**
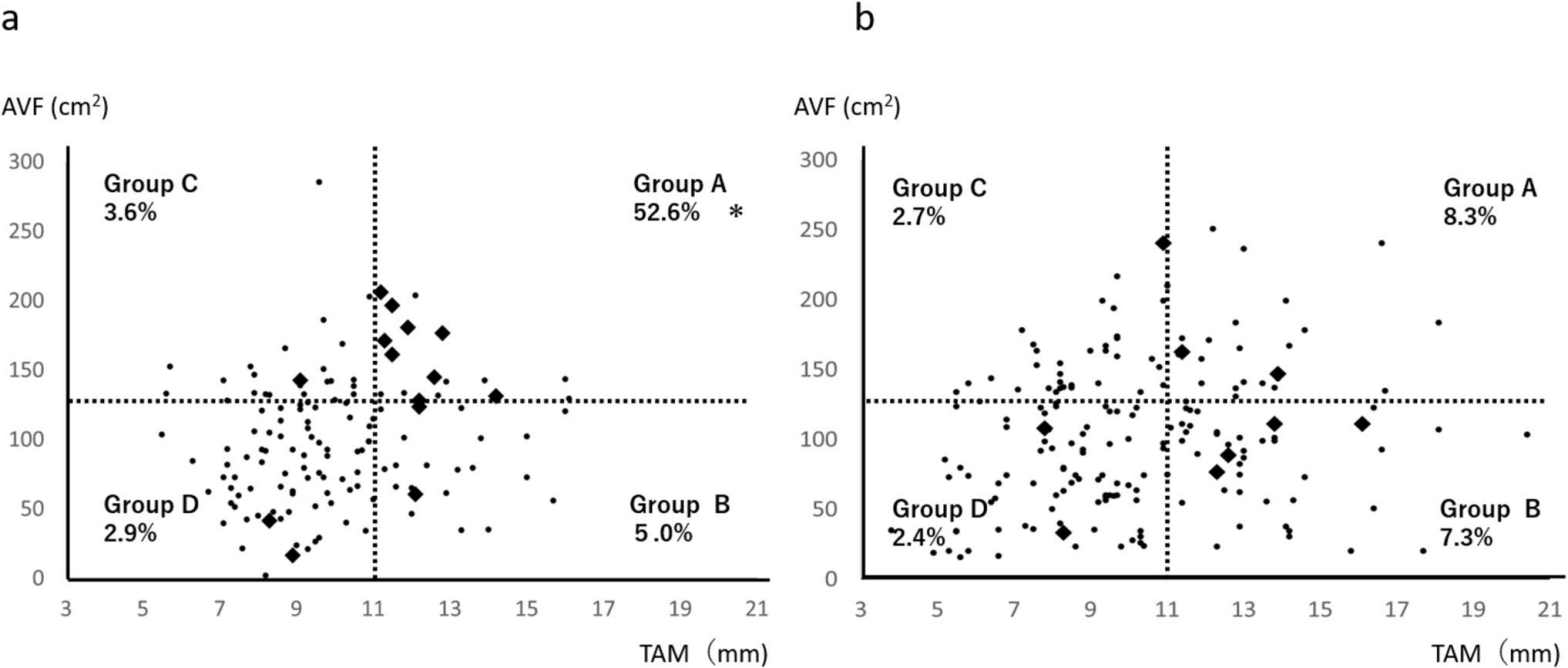
Scatter plot illustrating the relationship between TAM and AVF. **a** Control cohort. **b** Validation cohort. ◆: Outlet obstruction ( +); ●: outlet obstruction (−). *TAM* thickness of the rectus abdominis muscle, *AVF* area of the visceral fat

On the basis of these cutoffs, patients were stratified into four groups: group A (TAM ≥ 11.0 mm and AVF ≥ 123.0 cm^2^), group B (TAM ≥ 11.0 mm and AVF < 123.0 cm^2^), group C (TAM < 11.0 mm and AVF ≥ 123.0 cm^2^), and group D (TAM < 11.0 mm and AVF < 123.0 cm^2^). Outlet obstruction occurred in 52.6%, 5.0%, 3.6%, and 2.9% of patients in groups A, B, C, and D, respectively (Fig. 6A). Stratification effectively identified high-risk patients, with group A demonstrating a significantly higher incidence than all other groups (*p* < 0.001). The length of the fascial opening at the stoma site was measured using sagittal CT images obtained before stoma closure. The mean fascial length was 4.11 cm (SD 0.943) in the control cohort and 4.23 cm (SD 0.806) in the validation cohort (*p* = 0.681). Among high-risk patients (group A), the mean length was 4.51 cm (SD 0.706) in the control cohort and 4.75 cm (SD 0.712) in the validation cohort (*p* = 0.136).

In the validation cohort, outlet obstruction occurred in eight patients (4.4%), a significantly lower rate than in the control cohort (*p* = 0.047). By subgroup, outlet obstruction rates in the validation cohort were 8.3%, 7.3%, 2.4%, and 2.7% in groups A, B, C, and D, respectively (Fig. 6B). No significant differences were observed between cohorts in groups B, C, or D. However, group A demonstrated a significantly lower incidence in the validation cohort (*p* = 0.003) (Table 4).

**Table 4:** Incidence of outlet obstruction

Outlet obstruction (frequency [%])	Control group (*n* = 138)	Validation group (*n* = 180)	*p*-Value
All cases (*n* = 318)	15 (10.8%)	8 (4.4%)	0.047
Group A (*n* = 41)	9 (52.9%)	2 (8.3%)	0.003
Group B (*n* = 60)	1 (5.3%)	4 (9.6%)	0.783
Group C (*n* = 72)	2 (6.5%)	1 (2.4%)	0.574
Group D (*n* = 145)	3 (4.2%)	2 (2.7%)	0.677

## Discussion

In this study, increased TAM thickness and larger AVF were identified as independent risk factors for outlet obstruction. Furthermore, adjusting the rectus abdominis incision length significantly reduced the incidence of outlet obstruction. This study was not designed to compare surgical techniques, but rather to evaluate the clinical impact of a risk-based modification derived from anatomical predictors. Unlike previous studies that primarily focused on surgical techniques, this study emphasized patient-specific anatomical and metabolic factors. These findings underscore the importance of individualized loop ileostomy placement in high-risk patients. In this study, we excluded patients with a history of abdominal surgery or inflammatory bowel disease, including those who had undergone total colectomy. Prior abdominal surgery may lead to adhesions that reduce bowel mobility and potentially lower the risk of outlet obstruction. Moreover, total colectomy is associated with a higher incidence of outlet obstruction due to the lack of colonic fixation. These considerations allowed us to concentrate on a homogeneous population undergoing standard rectal cancer surgery with temporary loop ileostomy. To our knowledge, this is the first study to demonstrate that both AVF and TAM are independent, quantifiable predictors of outlet obstruction.

Advancements in surgical techniques and devices have enabled very low-level anastomoses. However, lower anastomoses are associated with an increased risk of AL [28]. Consequently, diverting stomas are routinely created to mitigate severe leakage and reduce the need for reoperation [3, 29]. Although diverting stomas offer these benefits, stoma-related complications, such as peristomal infection, stomal necrosis, dehydration due to high output, parastomal hernia, and outlet obstruction, remain significant concerns [30]. Despite their clinical relevance, few studies have evaluated the risk factors for outlet obstruction, and its pathophysiology remains incompletely understood.

Symptoms of outlet obstruction include abdominal distension, nausea, and vomiting. In most cases, decompression by tubing into the proximal limb of the ileostomy resolves the obstruction without the need for reoperation [14, 24]. Prior studies have reported potential risk factors for outlet obstruction, including total proctocolectomy, loop ileostomy configuration, tight fascial closure, ileostomy twisting, laparoscopic surgery, and thick TAM [9, 11, 12, 22]. Most of these are technical or procedural, with TAM thickness being the only previously identified patient-related factor. However, earlier studies did not explore the role of other body composition parameters, such as AVF, ASF, or TSF [22]. Identifying additional patient-related risk factors may enable the refinement of stoma construction techniques and optimization of outcomes in high-risk patients.

Outlet obstruction has been linked to caliber changes in the posterior layer of the rectus sheath at the ileostomy site. While previous reports identified TAM thickness as a contributing factor, this study provides a more comprehensive analysis by incorporating multiple measures of body composition. The data demonstrate that AVF, alongside TAM, is an important anatomical parameter that may affect stoma patency. We hypothesize that increased visceral fat may reduce bowel mobility and predispose the intestinal loop to torsion or compression at the stoma outlet. Additionally, the thickness and distribution of abdominal wall fat may influence the passage of the bowel through the fascial opening, potentially exacerbating narrowing due to postoperative edema. These factors may help explain the observed association between AVF and outlet obstruction. This hypothesis is supported by previous reports indicating that thick abdominal wall structures, including visceral and subcutaneous fat, may impair stoma mobility and contribute to outlet obstruction by increasing tension and narrowing at the fascial passage [31]. These anatomical constraints may exacerbate postoperative edema and reduce the effective lumen diameter at the stoma site.

Diverting stomas constructed after total proctocolectomy are particularly prone to outlet obstruction, likely due to the absence of colonic fixation [32], which increases the mobility and torsion susceptibility of the small intestine. In contrast, most procedures in this study were performed for conventional rectal cancer resections rather than for total proctectomy. The reported incidence of outlet obstruction following loop ileostomy after laparoscopic rectal resection, excluding total proctocolectomy, ranges from 5.4% to 27.3% [12, 21–23]. In the present study, the overall incidence was 7.2%, with 10.9% in the control cohort and 4.4% in the validation cohort. These rates are comparable to or lower than previously reported values.

Patients were stratified into four risk groups on the basis of the anatomical factors identified in this study. In the control cohort, the incidence of outlet obstruction was highest in group A (52.6%) and markedly lower in groups B, C, and D (5.0%, 3.6%, and 2.9%, respectively), compared with previously reported rates. In the validation cohort, incidence was similarly stratified, at 8.3%, 7.3%, 2.4%, and 2.7% in groups A, B, C, and D, respectively, demonstrating consistency and external validity. Although the overall incidence of outlet obstruction was relatively low (7.2%, 23/318), the control cohort included 138 patients, among whom occurred 15 cases of outlet obstruction (10.9%). For the multivariable logistic regression analysis, we limited the number of covariates to three (BMI, TAM, and AVF), selected a priori on the basis of clinical plausibility and univariate analysis (*p* < 0.1). This resulted in an events-per-variable (EPV) ratio of 5, which is below the commonly recommended threshold of 10. However, given the exploratory nature of the analysis and the careful selection of clinically relevant variables, we considered the analysis to be acceptable. The predictive performance of TAM and AVF was moderate (AUCs 0.73 and 0.724, respectively), and their combination enabled effective stratification of patients into clinically meaningful risk groups, with a high-risk group showing an incidence of 52.6%. Furthermore, the validation cohort confirmed the clinical relevance of these findings: the application of a modified stoma technique in the high-risk group significantly reduced the incidence of outlet obstruction to 8.3% (*p* = 0.003). This consistency across cohorts supports the robustness of the findings despite the modest number of events. Since the primary aim of this study was to identify anatomical risk factors rather than to develop a predictive model, the AUC values were presented as supplementary indicators to enhance the clinical interpretability of TAM and AVF in identifying high-risk patients. These findings reinforce that patients with both thick TAM and high AVF are at significantly higher risk for outlet obstruction. The identification of TAM and AVF as risk factors for outlet obstruction has notable clinical implications. Both parameters can be measured preoperatively using standard CT imaging, allowing for risk stratification prior to surgery. This allows surgeons to tailor the stoma construction technique for high-risk patients. In our validation cohort, the incidence of outlet obstruction was significantly reduced in patients with elevated TAM and AVF following the implementation of the modified technique. These findings highlight the value of incorporating anatomical metrics into preoperative planning to improve postoperative outcomes. In such patients, extending the rectus abdominis incision length or performing a controlled myotomy of the anterior sheath may reduce outlet obstruction rates.

The primary etiologies of outlet obstruction have been attributed to stenosis of the posterior rectus sheath and kinking of the bowel [11]. In this study, the risk from bowel torsion was minimal because the ileostomy was lifted naturally without rotation and was anchored to the anterior rectus sheath. No torsion was observed on postoperative imaging, and all cases of outlet obstruction resolved with decompression via tubing. These findings support the hypothesis that localized edema, rather than fixed mechanical obstruction, is a major contributor in anatomically predisposed individuals [22]. In particular, patients with thick TAM and large AVF may be more prone to passage narrowing due to postoperative edema in the abdominal wall.

In such high-risk cases, the conventional two-finger fascial opening may be inadequate, leading to a functional bottleneck. Although enlarging the fascial defect may lower obstruction risk, it may also increase the incidence of postoperative parastomal hernia. In the validation cohort, a modified technique, using only one finger’s width between the intestinal canal and fascia, was employed to optimize this balance. This approach significantly reduced outlet obstruction, especially in group A, and did not result in any clinically significant parastomal hernias. In this study, all patients underwent a temporary diverting ileostomy. Although the median follow-up period was 186 days (range 27–1090 days), approximately 90% of patients had their stoma closed within 10 months. Patients who received adjuvant chemotherapy tended to have their stoma closures delayed compared with others. To further evaluate the anatomical impact of the modified technique, we measured the length of the fascial opening at the stoma site on sagittal CT images obtained prior to stoma closure. The mean fascial length was 4.11 cm (SD 0.943) in the control cohort and 4.23 cm (SD 0.806) in the validation cohort (*p* = 0.681). Among high-risk patients (group A), the mean length was 4.51 cm (SD 0.706) in the control cohort and 4.75 cm (SD 0.712) in the validation cohort (*p* = 0.136). These findings suggest that the modified technique did not result in excessive fascial enlargement, even in high-risk patients, and support its anatomical safety. In this study, no patients needed surgical intervention for parastomal hernia. As part of routine clinical practice, all patients underwent CT imaging before their stoma closure, and no radiological evidence of clinically significant parastomal hernia was observed. Clinical follow-up did not reveal any symptomatic or surgically treated hernias. The modified technique was developed specifically for temporary stomas and is not intended for long-term diversion. Overall, the technique seems to be safe in the short term. Notably, no patients in either cohort required readmission for outlet obstruction within 30 days postoperatively, and all cases were managed during the initial hospitalization with uniform follow-up protocols. In addition to outlet obstruction, other stoma-related complications, such as parastomal hernia and high-output stoma, were observed in 8.5% and 17.6% of patients, respectively. However, the incidence of these complications did not differ significantly between the control and validation cohorts, suggesting that the modified ileostomy technique does not increase the risk of such issues. Taken together, these findings suggest that fascial modulation guided by patient-specific anatomical metrics, such as TAM and AVF, may improve stoma function while minimizing hernia risk. Given the simplicity and safety of this technique, it may be suitable for broader clinical adoption in high-risk patients.

Although this strategy showed promising results, the incidence of outlet obstruction did not differ significantly between low- and high-risk groups in the validation cohort, possibly due to the success of the modified approach. Several recent studies have proposed alternative methods to prevent outlet obstruction [32, 33]; however, further standardized prevention protocols remain lacking. Further investigation is warranted to refine risk stratification models and develop tailored prevention strategies that integrate patient-specific body composition.

Differences in robotic surgery rates, LLND frequency, and operative time between cohorts may reflect institutional trends, including expanded indications for robotic surgery, evolving criteria for LLND, and increased surgical experience. Importantly, in the control cohort, the incidence of outlet obstruction did not significantly vary on the basis of robotic approach, LLND status, or operative time, suggesting these factors have minimal influence on obstruction risk in this context. Although the study spans a long period, the stoma construction technique remained consistent, and procedural standardization was upheld across surgeons. Nevertheless, unmeasured confounding factors, such as metabolic indicators (e.g., blood glucose levels), may have influenced the outcomes. These parameters were not routinely recorded, and thus were not included in the analysis. We acknowledge this as a limitation and suggest that future studies incorporate such variables to enhance risk stratification.

This study has several limitations. First, it was conducted at a single institution, which may limit the generalizability of the findings. However, the consistent results observed in the validation cohort, particularly in risk stratification and the effectiveness of the modified stoma technique, suggest potential external validity. To confirm and expand upon these findings, future multicenter studies are warranted. Second, although the control cohort included 15 events among 138 patients, resulting in an EPV of 5, which is below the commonly recommended threshold of 10, we limited the number of covariates to three on the basis of clinical relevance. Given the exploratory nature of the analysis, we believe the model remains interpretable and informative. Third, the absence of a standardized definition of outlet obstruction complicates comparisons across studies. Finally, long-term evaluation of parastomal hernia development and other complications associated with larger myotomy incisions is required.

In conclusion, thick TAM and large AVF are independent, quantifiable anatomical risk factors for outlet obstruction after diverting loop ileostomy following laparoscopic and robotic rectal cancer surgery. The use of a modified stoma construction technique on the basis of these patient-specific metrics significantly reduced outlet obstruction without increasing hernia risk. These findings provide a novel, anatomy-based framework for surgical decision-making and underscore the need for multicenter validation to optimize and standardize preventative strategies.

## Data Availability

The datasets generated and analyzed during the current study are not publicly available due to patient privacy and institutional regulations. However, they are available from the corresponding author on reasonable request.
